# On-court throwing activity of male handball players during the European Championship 2020

**DOI:** 10.5114/biolsport.2023.116451

**Published:** 2022-07-21

**Authors:** Basilio Pueo, Juan Tortosa-Martínez, Luis Javier Chirosa-Rios, Carmen Manchado

**Affiliations:** 1 Physical Education and Sports, Faculty of Education, University of Alicante, 03690 Alicante, Spain; 2 Department of Physical Education and Sports, University of Granada, 18011 Granada, Spain

**Keywords:** Velocity, Effectiveness, LPS, Court, Goal, Heatmap

## Abstract

The aim of this study was to investigate the on-court throwing activity in regards to playing positions, throwing zones on the court and throwing velocity category during the male European Championship 2020. A local positioning system with microsensors placed both in the shirts of the players and inside the ball itself was used. In total, 6568 throws were retrieved for analysis from the entire tournament. Results showed that first-line players (wings and line players) used their natural zone more to throw (65% left wing, 60% right wing, and 97% line player), showing higher effectiveness from there (p < 0.05). Second lines players distributed more of their throws (45% left back, 50% right back and 32% center back in their natural zones) giving priority to the throws in 6 m, where they showed higher effectiveness (p < 0.05), or between the lines. Considering all players, shots from nearby and intermediate areas corresponded to 83% of the shots showing higher efficiency (p < 0.05) than shots performed from the areas furthest from the goal (14.9%. from zones 6-7-8). Back players mainly performed the highest velocity throws (Category 4 > ~100 km/h) from these furthest areas. Throwing velocity and effectiveness by throwing zones and positions was not significantly modified over the three rounds of the tournament (p > 0.05), indicating no effect of possible fatigue. A higher team ranking was associated with higher throwing efficiency but only for wing players. The results of this research could help handball coaches to better adjust training programs for the improvement of throwing velocity and its transfer to the competition.

## INTRODUCTION

Team handball is a fast-paced Olympic sport characterized by intermittent actions performed at maximal or near-maximal effort, interspersed with short recovery intervals [1, 2]. Among the fundamental movement skills in handball, such as running, blocking, jumping and throwing [3], the latter is of at most importance in goal actions [4]. Hence, throwing performance in handball is an essential factor to win a match [5].

Traditionally, handball analysis consisted of human observation during the matches, but results were constrained by visual observation and information processing limitations [6]. With the advent of audiovisual technology and the widespread low-cost and portable computers, data acquisition and analysis dramatically increased in accuracy and reliability [7]. Motion analysis of handball players has been acquired with optical systems, either single- [8, 9] or multiplecamera tracking systems installed on top of the court [10] for indoor venues, or with Global positioning systems (GPS) [11] for outdoor settings. Most studies using these tracking systems have been conducted either in controlled training conditions [4, 12, 13] or in national league games [14], providing limited information on the performance demands of handball at an elite level. In regards to the analysis of throwing velocity, several studies have attempted to measure this key variable in handball by using radar guns, photoelectric cells, and cinematography [13, 15–19]. However, most of these studies have been conducted without opposition or even a goalkeeper, in controlled training contexts. Only a few studies have analysed throwing velocity in real competition but without considering the position of the players or the court throwing zones [20].

More recently, local positioning systems (LPS) [21] have been introduced to give continuous positional tracking of both players and the ball in indoor venues. LPS is a radio-frequency based technology with high temporal and 3D spatial accuracy compared to errorfree criteria [22]. This instrument allows for a comprehensive analysis of the throwing action and positional data of both players and the ball in real competitions. The on-court activity of elite handball players in high-level championships is necessary for a better understanding of the game dynamics, especially concerning the throwing performance for playing positions and court throwing zones. To the knowledge of the authors, there is no available data defining the throwing activity on a court during an entire high-level tournament. Furthermore, the introduction of a microsensor inside the ball allows for obtaining higher precision data in regards to the speed of the ball, its location in the goal, the place from where it has been shot and the player who performed it.

Therefore, this study aimed to investigate the on-court throwing activity throughout the tournament in regards to playing positions, throwing zones on the court, throwing velocity categories, and according to the final ranking for top-level male handball players. To that end, an LPS system was used to acquire continuous positional tracking information of the players and the ball in the latest European Handball Federation (EHF) EURO 2020, held in Austria/Norway/ Sweden. This LPS system has been used in the Velux EHF Final4 and the first division of the German handball national league since the 2019/2020 season [23].

## MATERIALS AND METHODS

### Subjects

A total of 337 male players (age 27.8 ± 4.7 y, height 192.3 ± 6.7 cm, body mass 93.9 ± 11.8 kg and body mass index 52.3 ± 2.2 kg/m^2^) enrolled in 24 national teams taking part in the European Handball Federation (EHF) EURO 2020, held in Austria/Norway/Sweden, were included in this study. Player’s positions were identified according to the handball nomenclature: Left wing, LW (*n* = 49); Left back, LB (*n* = 66); Centre back, CB (*n* = 51); Right back, RB (*n* = 50); Right wing, RW (*n* = 44); and Line player, LP (*n* = 77). Goalkeepers were excluded from the analysis because their performance is not affected by the throwing characteristics. In total, the teams played 65 matches, from which 6568 throws were retrieved for analysis. The study was conducted according to the guidelines of the Declaration of Helsinki, and approved by the Ethics Committee of the University of Alicante (UA-2020-09-10).

### Instrumentation

The time-motion characteristics of players and the ball were collected through a LPS (Kinexon Precision Technologies, Munich, Germany), which has recently been validated against well-known systems such as GPS, showing proper between-device reliability (coefficient of variation around 5%) [22]. A complete description of the system can be found elsewhere [23]. The LPS can determine the real-time position and motion data of the player through a lightweight position chip (tag) positioned between shoulder blades using the manufacturer’s harness, whereas, for the ball, the same tag is incorporated in its centre. The sensor calculates 3D data (*x,y,z*) with position accuracy < 10 cm at a sampling frequency of 20 Hz for players and 50 Hz for the ball [22]. The position is determined using the time-of-flight (TOF) of ultra-wide-band radio signals travelling from the transmitter to the base stations, which calculate the actual 2D position of the tags within the playing field. Subsequently, instantaneous speed is derived by calculating the difference between two consecutive positions, i.e., approximating the derivative of the player or ball position. The raw position and speed data are filtered and smoothed using a Kalman filter for position data and an exponential moving average filter with a window length of 1 s for speed and position data. All data were analyzed using the system software (Kinexon Web Application, version 3.2.6, Munich, Germany).

### Procedures

This was a descriptive observational cross-sectional study to examine on-court throwing activity in regards to playing positions and throwing zones during elite competitive matches. The players were informed of the purposes, procedures, and risks and provided informed consent before the beginning of the study in a contract with the EHF. Personal data were anonymized for the purpose of this study. All the procedures were conducted in accordance with the Declaration of Helsinki and approved by the Ethics Committee of the University of Alicante (registration number UA-2020-09-10).

The following variables were extracted from the positional data of players and ball tags for each throw event. The absolute throwing velocity and categorized in velocity zones C1 (< 17 m/s), C2 (17–22 m/s), C3 (22–28 m/s), and C4 (> 28 m/s) were computed [24]. These zones correspond to approximately < 60, 60–80, 80–100, and < 100 km/h, respectively. The throwing position in the court was also categorized into eight zones within the court, [24]. In turn, these zones were also aggregated to account for the areas in which players perform throws to the goal as LW (1+2), LB (2+6), CB (3+7), RB (4+8), RW (4+5) and LP (2+3+4).

Likewise, effectiveness was calculated as the percentage relation between the number of throws that scored a goal and the number of throws, in accordance with similar handball studies [25, 26]. In order to check for differences in throwing velocity and effectiveness within a match, four equally timed periods of 15 min were categorized. Similarly, in order to study possible fatigue in the championship, the six finalist teams were compared in preliminary, main, and final rounds. Finally, the six finalist teams were compared to the teams ranked last in order to check for differences between ranked teams.

### Statistical Analyses

Descriptive data are presented as mean and standard deviation. The normality distribution of the data in all subgroups was checked through a Kolmogorov-Smirnov test. ANOVA tests were used to check differences between playing positions, throwing position zones, or velocity categories in regards to throwing velocity and effectiveness for the four periods within each match, the four stages of the tournament and the teams ranked first and last, followed by Games–Howell post hoc testing. Finally, a *z*-test was used to compare the proportion of goal, no goal and total throws when contrasted by each of the afore-mentioned groups. The alpha level of significance was set at *p* < 0.05. Statistical analysis was performed using the Statistical Package for Social Sciences (SPSS V22.0 for Windows, SPSS Inc, Chicago, USA).

## RESULTS

**FIG. 1:**
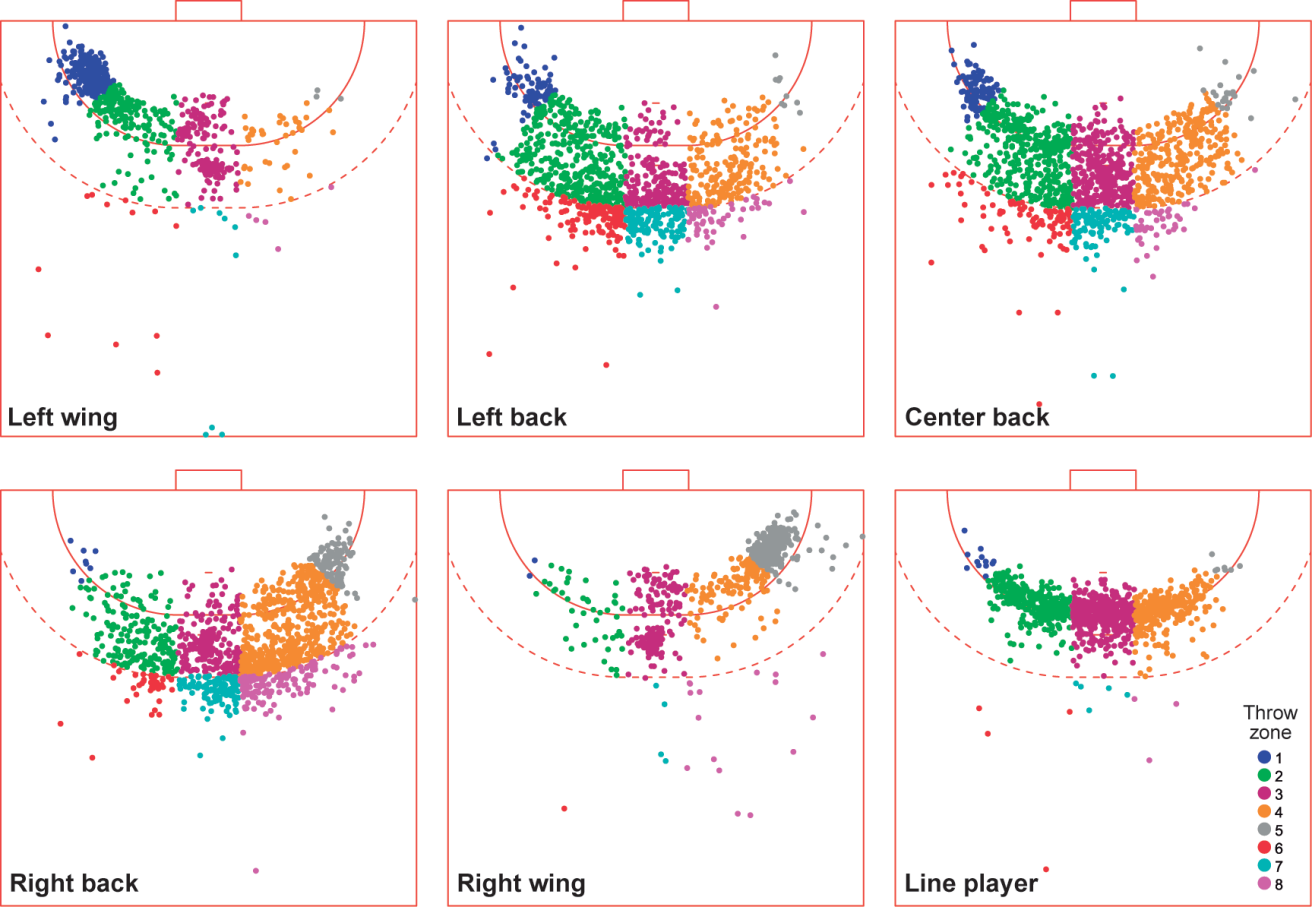
Heatmaps of throwing activity for different playing positions by throwing zone.

The distribution of throws in court for the six playing positions shows a marked difference in throwing activity for wing players, performing most actions inside zones 1, 2 for LW and 4, 5 for RW, as depicted in Figure 1. Contrastingly, back players execute more throws from adjacent positions than from their natural zones: LB and CB from zone 2 and RB from zone 4. Finally, CB shows a scattered distribution across central and side zones, whereas LP concentrates their throwing activity in the three central zones 2, 3 and 4.

Table 1 shows the throwing frequency activity split for throws resulting in a goal, no goal and total throws, calculated as the number of throws in a zone concerning the total for each playing position. LW showed high activity in zones 1 and 2 with more than 60% of all throws for the three results, where zone 1 was the most populated (*z* = 4.3–17.6, *p* < 0.05), followed by Zone 2 (*z* = 10.7–14.3, *p* < 0.05). Zone 3 was also very active, with one-quarter of the total throws (*z* = 10.0–13.7, *p* < 0.05). A similar trend can be observed for RW with zones 3, 4 and 5 (*z* = 4.2–14.8, 7.3–11.4, and 18.7–17.4, respectively, all *p* < 0.05). The activity of CB and LP is mostly performed in central zones 2, 3 and 4, with differences within zones (*z* = 2.4–4.2, *p* < 0.05) and between these zones and less populated ones (*z* = 2.4–18.0, *p* < 0.05). Finally, high throwing proportions in zones 2, 3 for LB and 3, 4 for RB were observed, with significant differences between them and the rest of the zones (*z* = 2.4–21.5, and 3.8–19.3, *p* < 0.05). As with wing players, LB and RB players perform in adjacent zones (4 and 2, respectively).

**TABLE 1:** Throw frequency (%) by playing positions and court throwing zones for goal, no goal and total throws.

		Throwing zones							

Pos.	Result	1	2	3	4	5	6	7	8
LW	Goal	35.0%*^(2,4–8)^ [152/434]	28.6%*^(1,4–8)^ [124/434]	29.3%*^(4–8)^ [127/434]	3.7%* [16/434]	0.5%*^(1–4)^ [2/434]	1.4%*^(1–4)^ [6/434]	1.4%*^(1–4)^ [6/434]	0.2%*^(1–4)^ [1/434]
	No goal	42.4%*^(2,4–8)^ [114/269]	24.5%*^(1,3–8)^ [66/269]	18.6%*^(2,4–8)^ [50/269]	9.3%* [25/269]	0.4%*^(1–4)^ [1/269]	2.2%*^(1–4)^ [6/269]	1.1%*^(1–4)^ [3/269]	1.5%*^(1–4)^ [4/269]
	Total	37.8%* [266/703]	27.0%*^(1,4–8)^ [190/703]	25.2%*^(1,4–8)^ [177/703]	5.8%* [41/703]	0.4%*^(1,4–6)^ [3/703]	1.7%*^(1–5)^ [12/703]	1.3%*^(1–4)^ [9/703]	0.7%*^(1–4)^ [5/703]

LB	Goal	6.0%*^(2–7)^ [34/565]	33.1%* [187/565]	18.2%*^(1,2,5–8)^ [103/565]	15.6%*^(1,2,5–8)^ [88/565]	0.4%* [2/565]	11.2%*^(1–5,8)^ [63/565]	11.5%*^(1–5,8)^ [65/565]	4.1%*^(2–7)^ [23/565]
	No goal	6.7%*^(2–7)^ [47/700]	32.3%* [226/700]	15.4%*^(1,2,5,7,8)^ [108/700]	15.7%*^(1,2,5,7,8)^ [110/700]	1.1%* [8/700]	13.3%*^(1,2,5,8)^ [93/700]	11.0%*^(1–5,8)^ [77/700]	4.4%*^(2–7)^ [31/700]
	Total	6.4%* [81/1265]	32.6%* [413/1265]	16.7%*^(1,2,5–8)^ [211/1265]	15.7%*^(1,2,5–8)^ [198/1265]	0.8%* [10/1265]	12.3%*^(1–5,8)^ [156/1265]	11.2%*^(1–5,8)^ [142/1265]	4.3%* [54/1265]

CB	Goal	4.5%*^(2–5,8)^ [29/641]	31.5%*^(1,4–8)^ [202/641]	28.4%*^(1,4–8)^ [182/641]	20.4%* [131/641]	0.9%*^(1–4,6,7)^ [6/641]	5.3%*^(2–5,8)^ [34/641]	6.7%*^(2–5,8)^ [43/641]	2.2%*^(1–4,6,7)^ [14/641]
	No goal	8.6%*^(2–5,8)^ [66/766]	26.0%*^(1,3,5–8)^ [199/766]	21.3%*^(1,2,5–8)^ [163/766]	22.5%*^(1,6–8)^ [172/766]	2.2%*^(1–4,6,7)^ [17/766]	7.4%*^(2–5,8)^ [57/766]	8.4%*^(2–5,8)^ [64/766]	3.7%*^(1–4,6,7)^ [28/766]
	Total	6.8%*^(2–5,8)^ [95/1407]	28.5%* [401/1407]	24.5%*^(1,2,5–8)^ [345/1407]	21.5%*^(1,2,5–8)^ [303/1407]	1.6%* [23/1407]	6.5%*^(2–5,8)^ [91/1407]	7.6%*^(2,5–8)^ [107/1407]	3.0%* [42/1407]

RB	Goal	0.7%*^(2–5,7,8)^ [4/603]	13.8%* [83/603]	20.2%* [122/603]	39.6%* [239/603]	6.1%*^(1–4,6,8)^ [37/603]	1.7%*^(2–5,7,8)^ [10/603]	8.6%*^(1–4,6)^ [52/603]	9.3%*^(1–6)^ [56/603]
	No goal	0.5%* [4/743]	14.7%*^(1,4–7)^ [109/743]	12.7%*^(1,4–6)^ [94/743]	37.8%* [281/743]	8.2%*^(1–4,6,8)^ [61/743]	3.9%* [29/743]	9.8%*^(1,2,4,6,8)^ [73/743]	12.4%*^(1,4–7)^ [92/743]
	Total	0.6%* [8/1346]	14.3%*^(1,4–8)^ [192/1346]	16.0%*^(1,4–8)^ [216/1346]	38.6%* [520/1346]	7.3%*^(1–4,6,8)^ [98/1346]	2.9%* [39/1346]	9.3%*^(1–4,6)^ [125/1346]	11.0%*^(1–6)^ [148/1346]

RW	Goal	0.2%*^(2–5,8)^ [1/470]	5.7%* [27/470]	36.2%*^(1,2,4,6–8)^ [170/470]	21.1%* [99/470]	34.5%*^(1,2,4,6–8)^ [162/470]	0.0%*^(2–5,8)^ [0/470]	0.2%*^(2–5,8)^ [1/470]	2.1%* [10/470]
	No goal	0.4%*^(2–5)^ [1/265]	6.8%* [18/265]	21.1%*^(1,2,5–8)^ [56/265]	21.5%*^(1,2,5–8)^ [57/265]	46.4%* [123/265]	0.4%*^(2–5)^ [1/265]	1.1%*^(2–5)^ [3/265]	2.3%*^(2–5)^ [6/265]
	Total	0.3%*^(2–5,8)^ [2/735]	6.1%* [45/735]	30.7%* [226/735]	21.2%* [156/735]	38.8%* [285/735]	0.1%*^(2–5,8)^ [1/735]	0.5%*^(2–5,8)^ [4/735]	2.2%* [16/735]

LP	Goal	0.7%*^(2–5)^ [4/572]	31.1%* [178/572]	41.1%* [237/572]	25.7%* [147/572]	0.0%*^(1–4)^ [0/572]	0.5%*^(2–4)^ [3/572]	0.2%*^(2–4)^ [1/572]	0.3%*^(2–4)^ [2/572]
	No goal	1.8%*^(2–4,6,8)^ [8/452]	30.1%*^(1,5–8)^ [136/452]	36.1%*^(1,4–8)^ [163/452]	29.2%*^(1,5–8)^ [132/452]	1.5%*^(2–4,6,8)^ [7/452]	0.2%*^(1–6)^ [1/452]	0.9%*^(2–4)^ [4/452]	0.2%*^(1–6)^ [1/452]
	Total	1.2%*^(2–4,6,8)^ [12/1025]	30.7%*^(1,3,5–8)^ [314/1025]	39.1%* [400/1025]	27.2%*^(1,3,5–8)^ [279/1025]	0.7%*^(2–4)^ [7/1025]	0.4%*^(1–4)^ [4/1025]	0.5%*^(2–4)^ [5/1025]	0.3%*^(1–4)^ [3/1025]

Note:

* Significance between zones for each position indicated by zone numbers in parenthesis. A number of throws for each zone out of the total for the position is indicated between brackets.

**FIG. 2:**
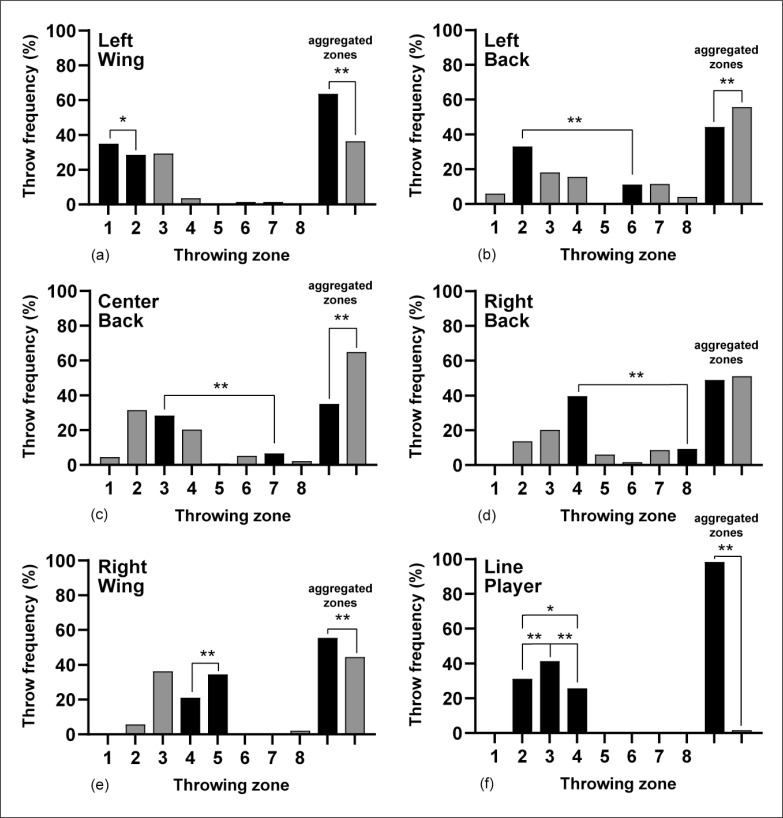
Throw frequency (%) by playing positions, court throwing zones and aggregated zones for throws resulting in goal. Note: * *p*<0.05, ** *p*<0.01: For clarity, significance is only indicated within zones in an aggregated zone and between aggregated zones.

The throwing activity for goal situations is depicted in Figure 2 for playing zones, together with zones aggregated according to the natural playing zones of players in a court (black) and the rest of the zones (grey). Significant differences were observed between aggregated zones for each playing position and the rest of the zones combined (*z* = 8.0, -3.9, -10.7, 3.4, and 32.6 for LW, LB, CB, RW, and LP, respectively, all *p* < 0.01), except for RB (*z* = -0.7, *p* = 0.45), which showed an overall throwing activity of 51.1% in zones other than 4 and 8. The other two playing positions with remarkable activity out of their natural playing zones were back players, which activity was 51.9% in zones 2 and 4 (CB), and 33.8% in zones 3 and 4 (LB).

A similar trend can be found for no goal and total throwing activity, as shown in Table 2. Differences were consistent both in percentage and significance for all positions (*z* = 3.4–32.6 for LW, RW, LP, and *z* = -3.3 – -18.9, *p* < 0.01), regardless of the throw result (goal, no goal and total), except for RB.

**TABLE 2:** Throw frequency (%) by playing positions and aggregated court throwing zones for goal, no goal and total throws.

Position (zones)	Left wing (1+2)	Left back (2+6)	Center back (3+7)	Right back (4+8)	Right wing (4+5)	Line player (2+3+4)
Goal	63.6%** [276/434]	44.2%** [250/565]	35.1%** [225/641]	48.9% [295/603]	55.5%** [261/470]	98.3%** [562/572]
No goal	66.9%** [180/269]	45.6%** [319/700]	29.6%** [227/766]	50.2% [373/743]	67.9%** [180/265]	95.4%** [431/452]
Total	64.9%** [456/703]	45.0%** [569/1265]	32.1%** [452/1407]	49.6% [668/1346]	60.0%** [441/735]	97.0%** [993/1024]

** *p* < 0.01: Significance between aggregated zones and the rest of the zones combined. A number of throws for each aggregated zone out of the total for the position indicated between brackets.

**FIG. 3:**
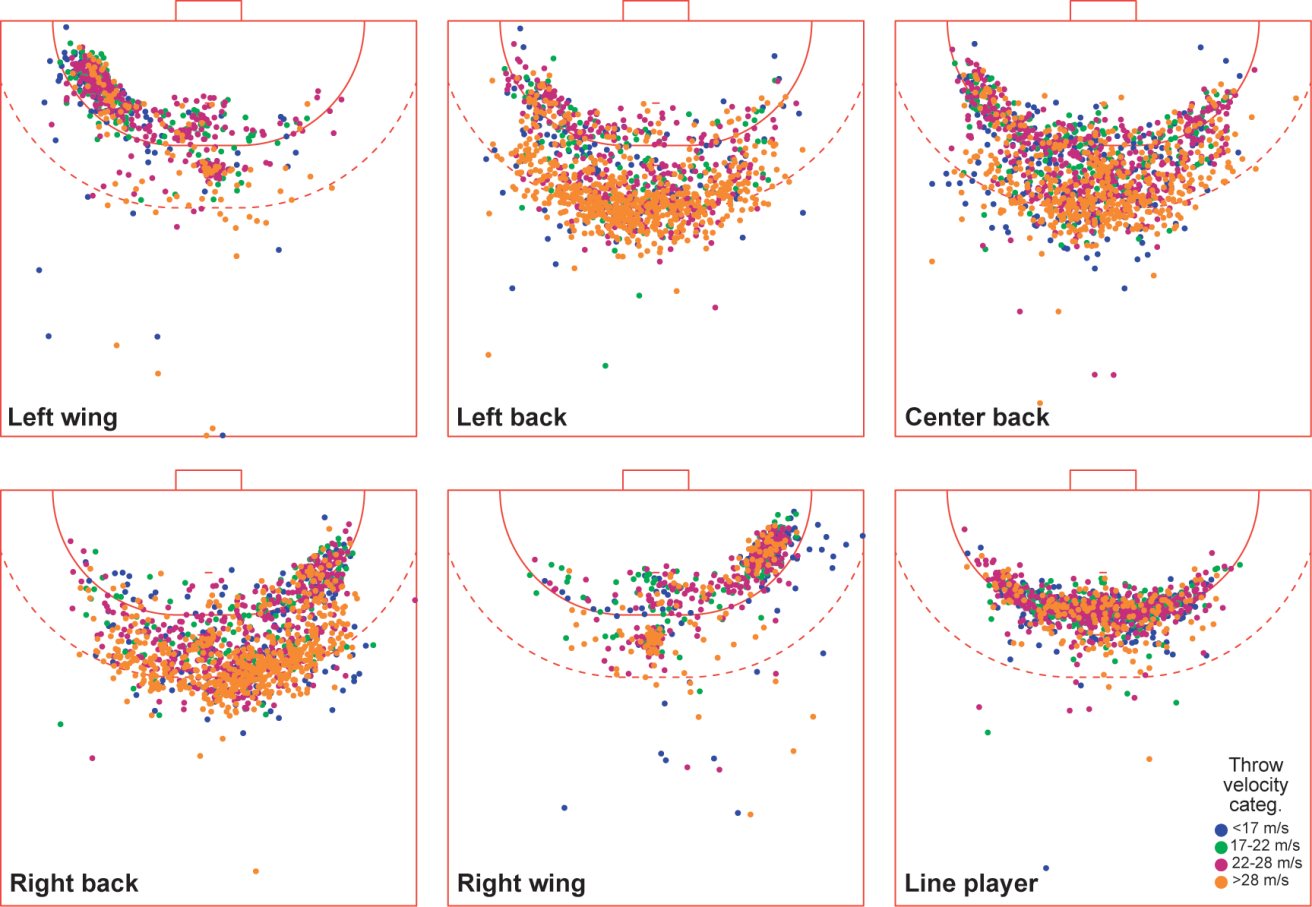
Heatmaps of throwing activity for different playing positions by velocity category.

Concerning throwing velocity, Figure 3 shows that most high-velocity throws in the C4 zone (> ~100 km/h) occur at large distances from the goal for the three back playing positions. Contrastingly, wing and line players use a variety of velocity zones regardless of throw distance.

**FIG. 4:**
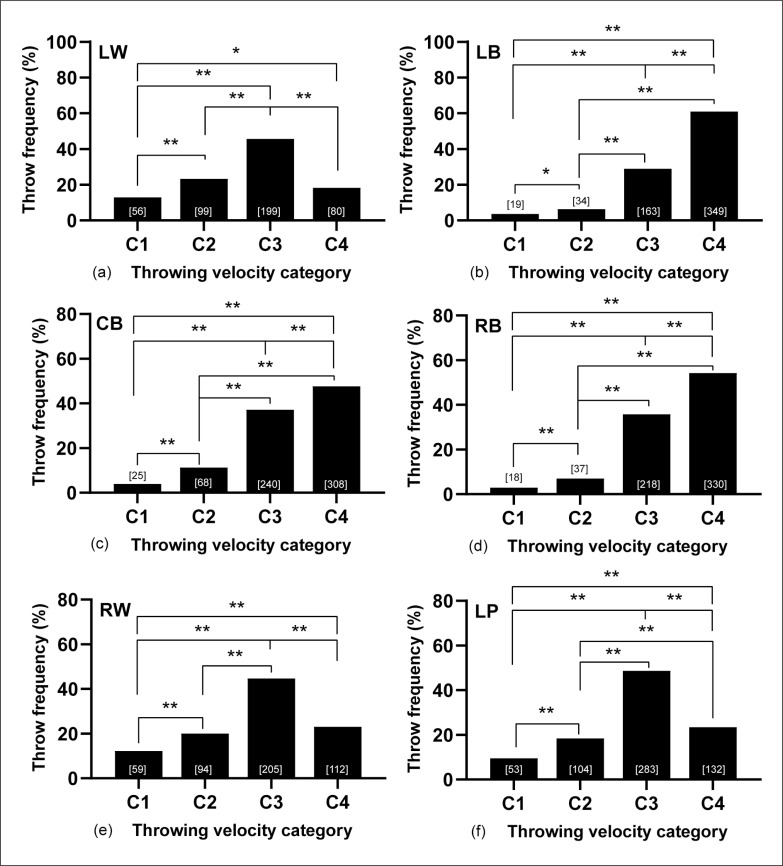
Throw frequency (%) by playing positions and velocity categories for throws resulting in goal. Number of throws indicated between brackets for each group. * *p*<0.05, ** *p*<0.01.

To study the influence of throwing velocity on playing positions for successful throws, Figure 4 depicts the differences in the four-velocity categories. The throwing activity for back players is very similar in terms of throw frequency and significant differences between the two lowest categories and C3 and C4 (*z* = 2.1–20.9, 4.6–18.0, and 2.6–19.8 for LB, CB and RB, respectively, all *p <* 0.05). For these playing positions, most of the throws are performed above ~80 km/h: 90.6% for LB, 85.4% for CB, and 90.9% for RB. Conversely, wing and LP showed a similar profile, by which around half of the throws are executed in the C3 zone: 45.9%, 43.6%, and 49.5% for LW, RW and LP, respectively. There were significant differences in successfully throwing activity in these playing positions between the lowest velocity category C1 and the rest (*z* = 2.2–14.9, *p* < 0.05), between C2 and C3 (*z* = 7.1–11.2, *p* < 0.05) and between C3 and C4 (*z* = 6.4–9.2, *p* < 0.05).

The throwing velocity and effectiveness by playing positions and court throwing zones according to four periods in a match are shown in Table 3. Consistent throwing velocity and effectiveness are observed across playing positions and therefore, ANOVA revealed no significant differences between periods in a match for throwing velocity (*F* = 0.81, *p* = 0.49), and for effectiveness (*F* = 0.53, *p* = 0.66). Similarly, ANOVA also indicated no differences for throwing zones between the four-match periods (*F* = 1.70, *p* = 0.16 for throwing velocity and *F* = 0.12, *p* = 0.95 for effectiveness).

**TABLE 3:** Throwing velocity (m/s) and effectiveness (%) by playing positions and court throwing zones according to four periods in a match, the three rounds in the championship and two rank-level teams.

	Position						Throwing zones							
	LW	LB	CB	RB	RW	LP	1	2	3	4	5	6	7	8
**Match**														

Period 1	23.1 ± 5.9	26.7 ± 7.0	24.4 ± 7.2	25.7 ± 7.3	22.8 ± 6.5	22.0 ± 7.2	22.0 ± 6.5	23.7 ± 7.5	24.5 ± 6.1	25.1 ± 7.2	21.7 ± 6.4	27.9 ± 8.6	27.7 ± 6.3	25.6 ± 8.7
	62 ± 49%	41 ± 49%	47 ± 50%	43 ± 50%	69 ± 46%	54 ± 50%	53 ± 50%	45 ± 50%	61 ± 49%	48 ± 50%	54 ± 50%	39 ± 49%	42 ± 49%	39 ± 49%
	[182]	[357]	[352]	[324]	[172]	[241]	[138]	[354]	[395]	[361]	[113]	[83]	[109]	[75]

Period 2	23.0 ± 5.6	26.7 ± 7.1	24.6 ± 7.3	26.7 ± 6.7	22.6 ± 5.9	21.4 ± 6.6	21.5 ± 5.8	24.7 ± 6.8	23.6 ± 6.6	24.9 ± 7.1	22.3 ± 5.9	26.5 ± 7.8	28.4 ± 7.1	28.3 ± 7.6
	55 ± 50%	44 ± 50%	45 ± 50%	46 ± 50%	64 ± 48%	55 ± 49%	42 ± 49%	50 ± 50%	58 ± 50%	51 ± 50%	47 ± 50%	37 ± 49%	46 ± 50%	42 ± 50%
	[184]	[329]	[390]	[350]	[212]	[262]	[124]	[440]	[402]	[399]	[119]	[83]	[94]	[66]

Period 3	21.6 ± 6.0	26.3 ± 7.0	24.5 ± 7.8	26.1 ± 7.0	22.6 ± 6.1	22.5 ± 6.8	21.3 ± 6.5	24.1 ± 7.1	24.2 ± 6.5	24.6 ± 7.0	20.2 ± 6.6	27.1 ± 7.8	29.2 ± 6.2	25.9 ± 8.9
	62 ± 49%	45 ± 50%	48 ± 50%	50 ± 50%	64 ± 48%	56 ± 50%	46 ± 50%	56 ± 50%	60 ± 49%	50 ± 50%	52 ± 50%	41 ± 50%	40 ± 49%	37 ± 49%
	[145]	[269]	[291]	[322]	[162]	[250]	[86]	[354]	[370]	[343]	[92]	[54]	[89]	[51]

Period 4	22.1 ± 6.0	26.6 ± 6.8	23.7 ± 7.8	25.4 ± 7.6	22.3 ± 6.1	23.0 ± 6.2	20.9 ± 6.6	23.7 ± 7.3	24.1 ± 6.3	24.5 ± 6.9	21.8 ± 6.3	26.2 ± 8.6	26.9 ± 7.5	26.8 ± 7.7
	67 ± 47%	49 ± 50%	42 ± 49%	41 ± 49%	59 ± 49%	58 ± 49%	51 ± 50%	55 ± 50%	61 ± 49%	44 ± 50%	43 ± 50%	37 ± 49%	43 ± 50%	40 ± 49%
	[192]	[310]	[374]	[350]	[189]	[271]	[116]	[407]	[408]	[394]	[102]	[83]	[100]	[76]

**Championship**														

Preliminary	22.7 ± 6.0	26.4 ± 6.6	24.8 ± 7.2	25.7 ± 7.6	23.3 ± 6.0	21.8 ± 6.3	21.6 ± 7.1	24.8 ± 6.7	23.5 ± 6.7	25.0 ± 6.5	22.4 ± 6.2	24.9 ± 9.9	26.2 ± 6.4	28.6 ± 7.4
	64 ± 48%	47 ± 50%	44 ± 50%	42 ± 50%	65 ± 48%	59 ± 50%	52 ± 51%	56 ± 50%	59 ± 49%	47 ± 50%	40 ± 49%	26 ± 44%	43 ± 50%	55 ± 50%
	[77]	[139]	[158]	[160]	[115]	[121]	[40]	[187]	[208]	[185]	[57]	[27]	[37]	[29]

Main	22.2 ± 5.9	26.2 ± 7.4	23.9 ± 7.6	25.3 ± 7.6	23.6 ± 5.9	22.5 ± 7.4	20.7 ± 7.0	23.8 ± 7.6	24.7 ± 6.5	24.6 ± 7.4	21.8 ± 6.9	25.8 ± 9.4	27.0 ± 8.4	27.0 ± 7.3
	71 ± 46%	47 ± 50%	46 ± 50%	49 ± 50%	73 ± 44%	56 ± 50%	55 ± 50%	50 ± 50%	60 ± 49%	56 ± 50%	56 ± 50%	33 ± 48%	42 ± 50%	48 ± 50%
	[85]	[180]	[232]	[244]	[132]	[172]	[56]	[273]	[269]	[260]	[75]	[36]	[47]	[29]

Finals	22.9 ± 6.1	26.2 ± 6.8	23.8 ± 8.2	25.8 ± 7.1	24.1 ± 5.2	21.0 ± 7.8	22.8 ± 6.0	22.8 ± 7.3	22.7 ± 6.6	24.9 ± 6.8	19.4 ± 6.0	27.6 ± 8.1	30.0 ± 6.2	24.5 ± 11.1
	65 ± 49%	51 ± 50%	36 ± 49%	38 ± 49%	57 ± 50%	58 ± 50%	50 ± 51%	43 ± 50%	57 ± 50%	43 ± 50%	50 ± 51%	44 ± 50%	36 ± 49%	36 ± 50%
	[23]	[47]	[104]	[94]	[44]	[67]	[18]	[82]	[98]	[90]	[24]	[25]	[28]	[14]

**Rank**														

Top	22.0 ± 5.9	26.7 ± 7.1	24.4 ± 7.7	25.7 ± 7.3	22.4 ± 6.6	22.1 ± 6.5	20.8 ± 6.9	23.8 ± 7.3	24.0 ± 6.5	24.9 ± 6.9	21.4 ± 6.7	26.3 ± 9.0	28.1 ± 6.6	27.3 ± 8.2
	65 ± 48%*	47 ± 50%	44 ± 50%	45 ± 50%	68 ± 47%*	57 ± 50%	52 ± 50%*	51 ± 50%	59 ± 49%	50 ± 50%*	49 ± 50%	37 ± 48%	42 ± 50%	48 ± 50%
	[242]	[422]	[627]	[608]	[328]	[433]	[158]	[640]	[703]	[629]	[181]	[109]	[144]	[96]

Low	22.9 ± 6.1	26.1 ± 7.0	23.1 ± 7.0	25.5 ± 7.1	21.9 ± 6.1	21.5 ± 7.1	21.7 ± 5.7	23.8 ± 7.4	23.8 ± 6.4	23.9 ± 7.1	21.0 ± 5.9	25.1 ± 8.5	27.2 ± 7.0	26.8 ± 8.0
	51 ± 50%*	43 ± 50%	40 ± 49%	44 ± 49%	57 ± 50%*	51 ± 50%	40 ± 49%*	49 ± 50%	55 ± 50%	42 ± 49%*	46 ± 50%	39 ± 49%	44 ± 50%	42 ± 50%
	[222]	[426]	[304]	[316]	[193]	[288]	[145]	[430]	[405]	[405]	[120]	[71]	[99]	[74]

* *p* < 0.05: Significance between top- and low-ranked groups. A number of throws for each group is indicated between brackets.

The throwing velocity and effectiveness of playing positions and court throwing zones are also shown in Table 3 for the three rounds of the tournament. Results indicated that both variables maintain little variations for playing positions across the tournament, and therefore, no statistical change is present, according to ANOVA (*F* = 0.89, *p* = 0.41 for throwing velocity and *F* = 2.0, *p* = 0.13 for effectiveness). In the same way, no differences were observed for throwing velocity (*F* = 0.13, *p* = 0.89) and effectiveness (*F* = 1.0, *p* = 0.37) in the activity performed in the court throwing zones.

Finally, the throwing velocity and effectiveness by playing positions and court throwing zones for the two rank-level teams are given in Table 3. While no differences were observed in throwing velocity by playing positions between top- and down-ranked teams (*F* = 3.15, *p* = 0.08), ANOVA showed significant differences in effectiveness (*F* = 16.27, *p <* 0.01, *η*^2^ = 0.04). Post hoc revealed that wing players were more efficient in top-ranked teams than in down-ranked teams: +13.6% for LW (*p <* 0.01), and +9.9% for RW (*p <* 0.05). Conversely, both throwing velocity and effectiveness showed consistency across the tournament when analyzed by throwing zones (*F* = 2.12, *p* = 0.15 and *F* = 3.72, *p* = 0.06, respectively).

## DISCUSSION

This study aimed to investigate the on-court throwing activity in regards to playing positions, throwing zones in the court and throwing velocity category in the matches played during the Men’s EHF EURO 2020. The introduction of a microsensor inside the ball itself has allowed analyzing in an ecological way variables related to the shots that had never been able to be studied directly (like combining the PP with the throwing zone) and even less in a major sporting event such as the EHF EURO 2020.

The most relevant data showed that first-line players (wings and LP) use their natural zone more to throw. A 65% of the LW, a 60% of the RW and a 97% of the LP’s throws were carried out from what is considered their playing position. Specificity is what prevails in these players. In the case of the second lines (LB-CB-RB) the throwing distribution from their playing position is not so high (45% for LB, 50% for RB and 32% for the CB) giving priority to the throws in 6 m or between the lines. The CB is the one that makes the closest throws to the 6 m line, almost 83% of its throws compared to 73% and 77% of the LB and RB respectively. The CB shows more diversity, with a slight tendency to finish in zone 2 and 3. The central player has a spatial advantage due to his position, to move in a variety of ways either in parallel or vertical to the defence and thus has more opportunities to throw from different positions [27]. As in most teams, the CB are right-handed, and they tend to come out to their weak point. The LB is the one who throws the most from a distance of all positions (28%).

The main difficulty of this research is to find studies in which the shots by position and areas are analyzed as is our case. The only study with a similar objective is that of Hatzimanouil [27], but they do so by analyzing a lower level of play and with a different orientation, comparing natural areas and positions with other areas and positions.

The effectiveness of a handball throw can be determined, among other factors, by distance to the goal. Blanco [28] and Almeida et al. [29], observed that proximity shots had a positive relationship with success compared to remote shots, where the relationship is negative. In our study, shots from nearby and intermediate areas correspond to 83% of the total of 6568 shots analyzed. The shots made from the areas furthest from the goal (zones 6-7-8) correspond to 14.9% of the total. Even back players, that are supposed to throw from long distances, showed a frequency of just 22.5%. The results are similar to those obtained by other studies [28, 30, 31], very far from the data provided by Eftene et al. [32] that estimate remote shots to be around 45.66%. These differences may be due to the registration system based on direct observation, compared to the registration through technological resources used in the present study.

Concerning effectiveness, first-line players (Wings and LP) showed higher effectiveness when throwing from their playing position than from aggregated zones. Curiously, LP’s effectiveness is slightly higher from zone 4 than 2, which is usually the weak side of right-handed players. Although second-line players are supposed to score from long distances, they showed higher effectiveness when throwing from aggregated zones close to the 6 m line, with exception of the RB who showed similar values, meaning a greater specialization than the LB.

One of the most relevant results of this study is that most of the shots that are made at high speed (zone C4) are executed from long distances from the goal (zone 6-7-8) and are mainly undertaken by the back players. In this sense, Tuquet et al. [31] indicate that a throw, when made from the middle distance (9 m) and between the lines (6–9 m), must be strong and powerful so that it can surprise the defence.

The differences in throwing velocity between the first and second lines could also be explained by anthropometric factors [33]. Studies in this regard show that second-line players are typically taller and larger [34], with a longer arm span that favours greater application of force, showing higher levels of muscle mass, strength and power [4].

Another relevant issue of this study is to verify that more than 80% of the throws in all positions are made at high or very high speeds (categories 3 and 4). In the same line, Tuquet et al. [31], who analyzed 1049 throws of a high-level men’s championship, found that more than 95% of the throws were made at the highest possible speed compared to only 5% that were ability shots. Regarding effectiveness, the wings are the most effective players with 62% and 64% (LW and RW respectively). The shots volume is over 50% lower than the back players, which are over 45% effective, with the CB being the one that makes a greater volume of throws. The data are similar to those obtained by Blanco [28], 66% for the wings and 49% for the RB and LB and much lower concerning the CB with 58%.

Analyzing throwing velocity, the first line players (wings and LP) showed greater efficiency in the shots category 3, while the second line players (backs) showed it in category 4. This fact could indicate that the backs should be lavished more on external shots, executed at maximum speed. This lower throwing velocity of the wings and LPS may be because many of the shots are close to goal, without opposition and contact. As this is an optimal condition for the shot, the effectiveness increases threefold [33]. Another of the aims of this study was to know if the throwing activity in terms of playing positions, throwing zones on the court and the speed category would be affected by the moment of the match, differentiating four quarters. The results showed that the time does not influence the variables analyzed. When analyzing if the fatigue accumulated throughout the championship could influence any of the variables analyzed, it was found that throwing velocity and effectiveness by throwing zones and positions did not show significant differences throughout the three rounds of the tournament. Possibly this result is because being a high-level event in which the best players from each country play, the coaches can rotate their players, allowing the variables analyzed not to be affected by fatigue.

Finally, regarding the study of situational variables analyzed in this study, such as the effect of ranking, it was observed that the wings of the best-classified teams were more efficient than those of the lower-ranked teams, coinciding with one of the predictive variables proposed by Almeida et al.[35].

These data are difficult to be compared with other research. The studies that have more similarities are those that compare winning teams with losers [36–39]. Their results showed that high-level teams were more efficient in all analyzed throwing related capabilities [37]. In our case, this only happens with the wings. The method of data collection may be an explanatory factor for the differences, together with the heterogeneity of the studies. The remarkable thing is that thanks to the LPS in this research, the data collection has been direct and automatic compared to the indirect methods of other studies. In this sense, the way to obtain data indirectly raises serious doubts about the reliability and validity of the registration [40].

The results of this research suggest that for the improvement of throwing performance, coaches should adjust training programs and loads to the conditions that will later be found in competition (zones, distance, opposition, etc.). This would probably allow minimizing the gap that exists between the improvement of the throwing velocity under training conditions and its transfer to the competition. We agree with Vila et al [25] that a study that analyzes the effect of increased throwing velocity in training on the throwing performance in matches should be carried out. For this, the use of microsensors inside the ball used in this research could be useful.

### Limitations

Although with LPS devices we can immediately measure the throwing activity of a championship, considering playing position, throwing zone, throwing velocity and location of the ball in the goal, the degree of opposition is not known automatically.

## CONCLUSIONS

The findings of this study revealed different throwing activities for playing positions in regards to the throwing zones and effectiveness on the court. First-line players showed a balanced throw distribution among zones, with scattered ball velocity and higher effectiveness in distances close to the goal, whereas second-line players used their natural zone more to throw with higher velocities. There was also no effect of fatigue in throwing activity throughout the tournament, which could be due to being elite players than can be changed on demand. Finally, only wing players showed higher throwing efficiency in higher ranking teams.
